# Passing the torch at *Brain Communications*

**DOI:** 10.1093/braincomms/fcag052

**Published:** 2026-03-03

**Authors:** Tara L Spires-Jones

**Affiliations:** Edinburgh, UK

## Abstract

The final editorial from our founding editor-in-chief introduces the new editor-in-chief, Professor David Belin from University of Cambridge, UK.

Welcome to Volume 8 issue 2 of *Brain Communications*. Our journal was launched 7 years ago, in 2019, as the sister journal to *Brain*. *Brain* was founded in 1878 by a group of neurologists and, from the outset, it has been a journal published by academics for academics. Originally, *Brain* was published by founding editors from the Neurological Society of London, but when this group became part of the Royal Society of Medicine in 1907, a new learned society, the Guarantors of *Brain*, was created to continue publishing the journal.^1^  *Brain* was the first major journal to focus on neurology; it later expanded to translational neuroscience and remains one of the most respected journals in the field. In the 21st century, the Guarantors decided (after much deliberation) to create a sister journal for *Brain* to begin to shift from the traditional paper magazine format that relies on institutional subscriptions towards the more modern online-only, open access model that is becoming dominant in scholarly publishing due to changes in the ways scientists access papers and changes in funding models and priorities of funders.

I was honoured in 2018 to be asked by the Guarantors to lead this new sister journal of *Brain* as its founding editor. This was my first experience as editor-in-chief for a journal and there was a steep learning curve, but it has been a privilege to build the journal around our ethos focusing on publishing rigorous, reproducible translational neuroscience studies and fostering an inclusive community of translational neuroscientists. I am proud of the growing reputation of the journal and the use of the funds we raise to support outstanding research fellows, travel grants and other initiatives that benefit translational neuroscientists. Now that my 7-year tenure as editor is ending, I am delighted to pass the torch of editor-in-chief to Professor David Belin.

David is professor of behavioural neuroscience at the University of Cambridge. He trained at the University of Bordeaux, in France, earning undergraduate and master’s degrees in molecular and cellular physiology and a PhD in neuroscience and neuropharmacology. During his PhD with Dr Véronique Deroche-Gamonet, he developed the first multidimensional preclinical model of cocaine addiction in the rat. He then moved to Cambridge for postdoctoral work with Professor Barry Everitt, studying psychobiological mechanisms of habitual and compulsive drug seeking and taking. Since 2009, David has led research groups at INSERM in France, and later in Cambridge, with a focus on understanding the neurobiology underlying the individual vulnerability to developing impulsive/compulsive spectrum disorders, including substance use disorder, alcohol use disorder and obsessive-compulsive disorder.

In addition to his research, David has been a strong advocate for the neuroscience community throughout his career. David was one of the founding scholars in the Federation of European Neuroscience (FENS)-Kavli network, contributing to supporting the careers of neuroscientists and advocating for neuroscience. Recently, David was elected as treasurer of the International Brain Research Organisation (IBRO), contributing to their mission to promote equitable and inclusive access to neuroscience education, training, research and engagement around the world. David has worked on editorial boards for several society journals, including as the translational neuroscience section editor of the *European Journal of Neuroscience*.

Closer to home, David has been an associate editor for *Brain Communications* since our inception, and his input has helped shape and drive our journal from the beginning. In one of our early editorial board meetings discussing the peer review process, David suggested we could contribute to constructive peer review in our field by training early career researchers in how best to review papers. This led us to start the Reviewer Academy in which David hosted an excellent training session on constructive peer review, which we still use to train new academy participants.^2^ David has published the first protocol paper of *Brain Communications*,^3^ and he has contributed to several editorials for our journal, including pieces on peer review,^4^ the advantages of publishing in society journals like ours^5^ and the problem with using a journal impact factor to judge the quality of papers published by it.^6^ I look forward to watching our journal flourish under his leadership.

Finally, I wish to thank our authors, reviewers, readers, the Guarantors and *Brain Communications* supporters for making the past 7 years so enjoyable as founding editor. A special thank you to our Associate Editors, Managing Editor Catriona Byres, Scientific Editor Manuela Marescotti and Publisher Phil Bishop, who have kept the journal running. I have learned a huge amount from all of you. Whilst I will miss being involved in the day-to-day running of the journal, I am delighted to stay on the team as an advisory editor, and look forward to having more spare time to fill the hour or two a day I have been dedicating to reading the fantastic papers submitted to *Brain Communications*.

The cover for this issue is based on work by Leckey, Giovannucci *et al*.,^7^ who developed an assay to measure proteins secreted by the choroid plexus and their clearance from cerebrospinal fluid. The image was created by Janis Malcomson, who was a patient at the UCLH National Hospital for Neurology and Neurosurgery, Queen Square, London.
